# Supplementary material for: Effects of the Fibrous Root of Polygonatum cyrtonema Hua on Growth Performance, Meat Quality, Immunity, Antioxidant Capacity, and Intestinal Morphology of White-Feathered Broilers
Source: Antibiotics (Basel). 2023 Nov 15;12(11):1627. doi: 10.3390/antibiotics12111627 (PMC10669470; doi:10.3390/antibiotics12111627)
Supplement: Supplementary file 1 [file antibiotics-12-01627-s001.zip › antibiotics-2656080-supplementary.pdf]

Table S1. Effects of the fibrous root of P. cyrtonema Hua on growth performance of white-feather.

Items	CON Group	ANT Group	LD Group	MD Group	HD Group	PR Group
**1~21d**						
Initial weight（g）	46	46	46	46	46	46
Final weight（g）	708.57±34.83^{Aa}	637.17±99.81^{ABab}	573.62±95.66^{ABbc}	511.30±96.62^{Bc}	552.31±51.58^{Bbc}	569.91±84.46^{ABbc}
ADG（g）	31.81±1.85^{Aa}	27.02±5.56^{ABab}	24.93±4.67^{ABbc}	20.78±5.59^{Bc}	23.64±2.91^{Bbc}	24.51±4.37^{ABbc}
ADFI（g）	42.59±2.57^{a}	41.29±8.39^{bc}	36.17±5.60^{bc}	34.14±6.44^{c}	36.35±4.84^{bc}	36.80±6.72^{bc}
F/G	1.34±0.05^{Bc}	1.54±0.51^{ABab}	1.47±0.17^{ABbc}	1.67±0.14^{Aa}	1.54±0.08^{ABab}	1.50±0.12^{ABabc}
**22~42d**						
Final weight（g）	2476.67±70.90^{ABa}	2712.08±321.42^{Aa}	2559.49±108.56^{ABab}	2275.04±215.72^{Bc}	2350.89±158.60^{Bbc}	2541.91±237.20^{ABab}
ADG（g）	83.70±3.21^{Bb}	97.80±12.10^{Aa}	91.11±4.08^{ABab}	83.96±6.90^{Bb}	84.86±6.57^{ABbc}	92.81±10.26^{ABab}
ADFI（g）	134.03±11.67^{ab}	145.68±16.01^{a}	130.74±16.26^{ab}	126.56±14.18^{b}	129.85±12.16^{ab}	140.00±13.05^{ab}
F/G	1.60±0.16	1.50±0.15	1.43±0.16	1.51±0.18	1.53±0.16	1.52±0.14
**1~42d**						
ADG（g）	63.30±3.66^{Aa}	63.48±7.65^{Aa}	59.85±2.58^{ABab}	53.07±5.14^{Bc}	54.88±3.78^{ABbc}	59.43±5.65^{ABab}
ADFI（g）	88.31±6.86	93.48±9.37	80.60±10.73	88.55±15.47	83.38±5.61	88.84±9.39
F/G	1.39±0.07^{Bb}	1.48±0.08^{ABb}	1.35±0.19^{Bb}	1.67±0.22^{Aa}	1.52±0.13^{ABab}	1.50±0.09^{ABab}

Note: In the same row，values with different small letter superscripts mean significant difference (P＜0.05)，and with different capital letter superscripts mean significant difference (*P*＜0.01)，while with the same or no letter superscripts mean no significant difference (*P*＞0.05).

Table S2. Effects of the fibrous root of P. cyrtonema Hua on slaughter performance of white-feather broilers.

Items	CON Group	ANT Group	LD Group	MD Group	HD Group	PR Group
Dressing percentage	90.57±2.36^{B}	91.30±1.89^{B}	94.23±2.05^{A}	95.14±1.28^{A}	95.63±1.70^{A}	94.97±1.32^{A}
Semi-eviscerated rate	82.19±3.34^{b}	83.93±3.14^{ab}	85.09±2.12^{ab}	86.25±1.52^{a}	86.25±1.38^{a}	84.87±2.98^{ab}
Eviscerated rate	71.61±3.62	72.16±2.80	73.68±2.08	74.28±1.45	74.45±1.04	73.48±2.33
Breast muscle rate	22.18±1.68	21.26±1.34	22.68±1.79	22.72±1.11	22.88±1.09	20.91±2.39
Thigh muscle rate	14.65±0.91^{ab}	13.59±0.87^{ab}	14.16±1.83^{ab}	14.35±0.99^{ab}	13.41±1.91^{b}	15.45±1.08^{a}
Abdominal fat rate	1.52±0.46^{Aa}	0.74±0.26^{Bb}	1.01±0.49^{ABb}	1.03±0.32^{ABb}	0.64±0.33^{Bb}	0.91±0.25^{ABb}

Note: In the same row， values with different small letter superscripts mean significant difference (P＜0.05)， and with different capital letter superscripts mean significant difference (*P*＜0.01)， while with the same or no letter superscripts mean no significant difference (*P*＞0.05)

Table S3. Effects of the fibrous root of P. cyrtonema Hua on leg muscle of white-feather broilers.

Items	CON Group	ANT Group	LD Group	MD Group	HD Group	PR Group
L*	52.57±5.54	49.13±7.66	51.41±3.48	53.32±4.37	49.66±4.16	49.64±2.14
a*	17.00±4.48^{ab}	18.77±4.36^{ab}	18.84±1.69^{ab}	16.80±2.41^{b}	19.67±2.31^{Ab}	21.18±2.79^{a}
b*	11.22±3.18	11.20±1.98	10.73±1.13	9.64±1.74	10.50±1.31	11.13±1.73
pH45min	6.29±0.33	6.50±0.16	6.34±0.28	6.30±0.18	6.46±0.16	6.28±0.09
pH24h	5.74±0.39^{b}	6.00±0.16^{ab}	6.02±0.09^{a}	6.10±0.22^{a}	6.06±0.16^{a}	5.97±0.04^{ab}
pH reduction	0.55±0.52	0.49±0.10	0.32±0.26	0.33±0.21	0.40±0.15	0.30±0.06
Shear force/N	25.38±3.78^{Bb}	41.68±15.95^{ABa}	40.42±8.61^{ABa}	42.12±9.90^{ABa}	47.33±10.03^{Aa}	37.81±10.64^{ABa}
Drip loss	11.65±6.7^{ab}	12.38±3.83^{a}	10.38±9.94^{ab}	4.35±2.40^{b}	4.64±1.27^{b}	9.34±5.58^{ab}
Pressure loss	11.40±6.16^{a}	9.87±5.00^{a}	10.57±2.35^{a}	8.61±6.76^{ab}	5.56±2.47^{b}	11.37±3.96^{a}

Note: In the same row， values with different small letter superscripts mean significant difference (P＜0.05)， and with different capital letter superscripts mean significant difference (*P*＜0.01)， while with the same or no letter superscripts mean no significant difference (*P*＞0.05).

Table S4. Effects of the fibrous root of P. cyrtonema Hua on breast muscle of white-feather broilers.

Items	CON Group	ANT Group	LD Group	MD Group	HD Group	PR Group
L*	52.06±4.87	51.70±5.20	51.13±3.70	52.61±2.61	53.69±1.98	52.01±4.35
a*	14. 15±5.32	20.80±10.26	16.86±5.57	21.70±5.70	20.12±4.22	21.65±4.19
b*	$9.03±1.61^{b}$	$13.42±4.28^{a}$	$10.24±3.33^{ab}$	$11.86±2.04^{ab}$	$11.39±1.83^{ab}$	$12.29±2.16^{ab}$
pH45min	6.26±0.46	6.45±0.37	6.45±0.25	6.53±0.09	6.54±0.23	6.41±0.19
pH24h	5.77±0.12	5.82±0.16	5.84±0.15	5.81±0.11	5.84±0.09	5.81±0.10
pH reduction	0.49±0.43	0.63±0.40	0.62±0.29	0.71±0.13	0.69±0.26	0.60±0.22
Shear force/N	25.44±9.94	33.20±13.64	35.02±5.75	32.10±6.57	35.62±15.90	35.92±15.66
Drip loss	$9.00±3.93^{ABabc}$	$12.02±3.38^{ABab}$	$17.44±13.71^{Aa}$	$2.17±1.60^{Bc}$	$4.49±0.83^{Bbc}$	$9.67±6.05^{ABabc}$
Pressure loss	$9.86±4.98^{ab}$	$8.81±4.11^{ab}$	$11.94±5.72^{a}$	$5.00±3.73^{b}$	$4.94±2.41^{b}$	$12.40±6.38^{a}$

Note: In the same row, values with different small letter superscripts mean significant difference ($P<0.05$), and with different capital letter superscripts mean significant difference ($P<0.01$), while with the same or no letter superscripts mean no significant difference ($P>0.05$).

Table S5. Effects of the fibrous root of P. cyrtonema Hua on immune organ index of white-feather broilers.

Items	CON Group	ANT Group	LD Group	MD Group	HD Group	PR Group
**0~21d**						
Liver	26.54±2.50	29.54±3.38	27.48±2.83	26.73±7.10	27.64±2.01	27.42±1.92
Thymus	$3.32±0.76^{ab}$	$3.52±1.27^{ab}$	$2.79±0.59^{b}$	$3.94±1.06^{ab}$	$4.16±0.40^{a}$	$3.33±0.97^{ab}$
Spleen	0.78±0.24	1.01±0.38	1.07±0.47	0.73±0.10	1.03±0.20	0.99±0.18
Bursa	2.27±0.66	1.74±0.24	2.24±0.75	2.26±1.11	2.13±0.53	2.36±0.50
**22~42d**						
Liver	20.53±2.63	19.07±2.02	20.90±2.66	19.83±3.08	22.27±2.27	21.50±4.55
Thymus	$1.34±1.02^{Bc}$	$2.39±0.78^{ABabc}$	$3.47±1.28^{Aa}$	$2.76±1.11^{ABab}$	$1.93±0.89^{ABbc}$	$2.14±0.69^{ABbc}$
Spleen	1.19±0.44	1.40±0.51	1.24±0.18	1.36±0.24	1.65±0.72	1.61±0.71
Bursa	$0.63±0.29^{b}$	$0.69±0.21^{b}$	$0.66±0.24^{b}$	$0.63±0.14^{b}$	$1.16±0.73^{a}$	$0.67±0.18^{b}$

Note: In the same row, values with different small letter superscripts mean significant difference ($P<0.05$), and with different capital letter superscripts mean significant difference ($P<0.01$), while with the same or no letter superscripts mean no significant difference ($P>0.05$).

Table S6. Effects of the fibrous root of P. cyrtonema Hua on immunoglobulin levels in white-feather.

Items	CON Group	ANT Group	LD Group	MD Group	HD Group	PR Group
**1~21d**						
IgG (μg/mL)	63.20±3.77^{A}	54.65±3.17^{B}	54.90±3.40^{B}	48.35±5.04^{C}	61.96±3.87^{A}	56.83±3.51^{B}
IgM (ng/mL)	2544.77±184.00^{Bc}	2663.69±184.02^{Bbc}	2552.57±123.20^{Bc}	3122.16±227.34^{Aa}	2870.51±158.54^{ABb}	2659.01±279.02^{Bbc}
IgA (ng/mL）	7936.40±396.41^{B}	8894.81±492.69^{A}	8754.30±462.51^{A}	7482.38±401.43^{B}	6455.75±350.24^{C}	8967.50±322.62^{A}
C3 (μg/mL)	804.88±37.57^{A}	768.10±29.70^{A}	671.56±36.36^{B}	810.09±39.57^{A}	777.67±28.35^{A}	785.36±34.30^{A}
C4 (μg/mL)	431.24±20.01^{CDd}	454.43±11.62^{BCcd}	480.50±23.96^{ABbc}	515.00±18.53^{Aa}	401.03±25.94^{De}	485.13±26.08^{ABb}
**22~42d**						
IgG (μg/mL)	76.61±4.27^{D}	63.70±3.43^{E}	83.60±4.06^{C}	93.52±2.84^{B}	103.47±3.90^{A}	100.03±4.92^{A}
IgM (ng/mL)	4429.94±256.11^{Ab}	3098.34±238.85^{Bc}	4554.18±236.18^{Aab}	4654.23±239.74^{Aab}	4783.50±196.50^{Aa}	4712.24±177.53^{Aab}
IgA (ng/mL）	9438.17±163.53^{ABb}	9789.05±240.38^{Aa}	9281.64±335.69^{Bb}	8466.90±223.08^{Cc}	8647.87±368.56^{Cc}	8314.88±300.74^{Cc}
C3 (μg/mL)	847.80±35.13^{ABb}	865.49±13.91^{Aab}	795.98±40.14^{Bc}	900.88±46.10^{Aa}	881.62±38.15^{Aab}	860.32±41.10^{Aab}
C4 (μg/mL)	578.89±31.99^{ABa}	572.60±27.10^{ABa}	587.90±20.31^{Aa}	541.35±23.53^{Bb}	600.77±28.48^{Aa}	600.56±15.00^{Aa}

Note: In the same row，values with different small letter superscripts mean significant difference ($P<0.05$)，and with different capital letter superscripts mean significant difference ($P<0.01$)，while with the same or no letter superscripts mean no significant difference ($P>0.05$).

Table S7. Effects of the fibrous root of P. cyrtonema Hua on mRNA expression levels of cytokines in serum of white-feather broilers.

Items	CON Group	ANT Group	LD Group	MD Group	HD Group	PR Group
**1~21d**						
IL-2	1.37 ± 1.09^{Bb}	2.90 ± 2.17^{Aa}	0.21 ± 0.18^{Bbc}	0.15 ± 0.06^{Bc}	0.10 ± 0.02^{Bc}	0.52 ± 0.20^{Bbc}
IFN-γ	1.40 ± 1.22^{ABb}	2.62 ± 2.19^{Aa}	0.19 ± 0.13^{Bbc}	0.19 ± 0.10^{Bbc}	0.12 ± 0.05^{Bc}	0.53 ± 0.26^{Bbc}
**22~42d**						
IL-2	1.18 ± 0.78^{BCc}	1.49 ± 0.72^{BCc}	0.62 ± 0.39^{Cc}	4.41 ± 3.19^{Aa}	3.54 ± 1.62^{ABab}	2.27 ± 1.02^{ABCbc}
IFN-γ	1.18 ± 0.79^{BCc}	1.63 ± 0.82^{BCbc}	0.59 ± 0.39^{Cc}	4.42 ± 2.90^{Aa}	3.40 ± 1.78^{ABab}	2.00 ± 0.69^{BCbc}

Note: In the same row， values with different small letter superscripts mean significant difference (P＜0.05)， and with different capital letter superscripts mean significant difference (*P*＜0.01)，while with the same or no letter superscripts mean no significantd ifference (*P*＞0.05).

Table S8. Effects of the fibrous root of P. cyrtonema Hua on antioxidant function of white－feather broilers.

Items	CON Group	ANT Group	LD Group	MD Group	HD Group	PR Group
**1~21d**						
T-AOC (U/mL)	0.72 ± 0.10^{Aa}	0.72 ± 0.12^{Aa}	0.65 ± 0.07^{ABab}	0.58 ± 0.07^{Bbc}	0.55 ± 0.05^{Bc}	0.57 ± 0.07^{Bbc}
SOD (U/mL)	85.29±5.38	91.53±4.01	89.57±2.86	87.19±8.74	87.33±6.55	89.40±6.98
MDA (U/mL)	12.58 ± 2.50^{B}	22.48 ± 4.07^{A}	4.99 ± 0.82^{C}	6.38 ± 1.59^{C}	4.46 ± 0.82^{C}	5.10 ± 1.11^{C}
GSH-Px (U/mL)	39.38 ± 4.18^{B}	40.69 ± 3.68^{B}	39.13 ± 3.36^{B}	38.09 ± 4.15^{B}	47.39 ± 4.59^{A}	39.19 ± 2.92^{B}
**22~42d**						
T-AOC (U/mL)	0.85 ± 0.06^{ABab}	0.93 ± 0.04^{Aa}	0.85 ± 0.02^{ABab}	0.77 ± 0.12^{Bb}	0.85 ± 0.05^{ABab}	0.82 ± 0.08^{ABb}
SOD (U/mL)	76.97±6.76	80.38±13.18	76.40±5.81	79.76±3.14	81.56±9.90	79.75±4.99
MDA (nmol/mL)	7.88 ± 1.82^{C}	30.37 ± 2.68^{A}	15.15 ± 5.50^{B}	12.30 ± 8.90^{B}	12.11 ± 3.80^{B}	16.73 ± 4.60^{B}
GSH-Px (U/mL)	26.11 ± 2.48^{B}	34.06 ± 4.17^{A}	31.02 ± 0.52^{A}	25.30 ± 3.86^{B}	33.45 ± 2.79^{A}	34.27 ± 1.67^{A}

Note: In the same row， values with different small letter superscripts mean significant difference (P＜0.05)， and with different capital letter superscripts mean significant difference (*P*＜0.01)， while with the same or no letter superscripts mean no significant difference (*P*＞0.05).

Table S9. Effects of the fibrous root of *P. cyrtonema* Hua on intestinal morphology of white-feather broilers.

Items	CON Group	ANT Group	LD Group	MD Group	HD Group	PR Group
**1~21d**						
VH	937.98±196.54^{b}	930.34±130.80^{b}	1083.30±65.23^{ab}	1282.60±295.95^{a}	1179.47±215.31^{ab}	1075.23±213.74^{ab}
CD	217.11±29.46^{ABb}	218.80±38.49^{ABb}	270.40±32.21^{Aa}	226.97±32.63^{ABb}	224.44±44.47^{ABb}	205.19±35.94^{Bb}
V/C	4.33±0.70^{ABbc}	4.29±0.49^{ABbc}	4.05±0.48^{Bc}	5.63±0.95^{Aa}	5.37±1.13^{ABa}	5.26±0.78^{ABab}
**22~42d**						
VH	1038.94±109.00^{b}	1220.22±203.99^{ab}	1138.63±177.49^{ab}	1333.47±195.97^{a}	1362.66±146.43^{a}	1255.72±310.82^{ab}
CD	221.97±22.22^{b}	245.17±18.80^{ab}	227.35±48.02^{ab}	245.68±38.10^{ab}	267.30±32.36^{a}	210.33±21.92^{b}
V/C	4.71±0.61	5.05±1.21	5.13±1.00	5.50±0.93	5.14±0.63	6.41±1.32

Note: In the same row, values with different small letter superscripts mean significant difference (P＜0.05)，and with different capital letter superscripts mean significant difference (P＜0.01)，while with the same or no letter superscripts mean no significant difference (P＞0.05).
